# The Crosstalk Between Cell Adhesion and Cancer Metabolism

**DOI:** 10.3390/ijms20081933

**Published:** 2019-04-19

**Authors:** Bárbara Sousa, Joana Pereira, Joana Paredes

**Affiliations:** 1 Ipatimup-Institute of Molecular Pathology and Immunology of the University of Porto, 4200-135 Porto, Portugal; bsousa@ipatimup.pt (B.S.); jspereira@ipatimup.pt (J.P.); 2 i3S, Institute of Investigation and Innovation in Health, 4200-135 Porto, Portugal; 3 Medical Faculty of the University of Porto, 4200-135 Porto, Portugal

**Keywords:** metabolism, cancer, adhesion, cadherin, ECM, cancer stem cells

## Abstract

Cancer cells preferentially use aerobic glycolysis over mitochondria oxidative phosphorylation for energy production, and this metabolic reprogramming is currently recognized as a hallmark of cancer. Oncogenic signaling frequently converges with this metabolic shift, increasing cancer cells’ ability to produce building blocks and energy, as well as to maintain redox homeostasis. Alterations in cell–cell and cell–extracellular matrix (ECM) adhesion promote cancer cell invasion, intravasation, anchorage-independent survival in circulation, and extravasation, as well as homing in a distant organ. Importantly, during this multi-step metastatic process, cells need to induce metabolic rewiring, in order to produce the energy needed, as well as to impair oxidative stress. Although the individual implications of adhesion molecules and metabolic reprogramming in cancer have been widely explored over the years, the crosstalk between cell adhesion molecular machinery and metabolic pathways is far from being clearly understood, in both normal and cancer contexts. This review summarizes our understanding about the influence of cell–cell and cell–matrix adhesion in the metabolic behavior of cancer cells, with a special focus concerning the role of classical cadherins, such as Epithelial (E)-cadherin and Placental (P)-cadherin.

## 1. Introduction

Alterations in both cell–cell and cell–matrix adhesion occur along the multi-step cascade of cancer progression, allowing cancer cells to become more motile, to degrade the extracellular matrix (ECM), to survive in circulation, and to colonize distant metastatic sites. Importantly, physical alterations occurring in the ECM and in cancer cell mechanics require metabolic rewiring along this process to satisfy cancer cell’s energetic needs. In fact, the ability to modulate the cellular cytoskeleton and to reprogram cell metabolism are hallmarks of epithelial cancers. 

Cell–cell adhesion is mainly mediated by cadherins, while the contact with the ECM is mostly performed by integrins. These molecules largely contribute to cell signaling, growth, differentiation, inflammation, and survival. Although there are in silico, in vitro, and in vivo studies disclosing the link between cell adhesion molecules and the metabolic behavior in different types of cancer, it is far from being clearly understood. This review summarizes our understanding about the influence of cell–cell and cell–matrix interactions in cancer metabolism, with a special focus on classical cadherins, namely Epithelial (E)- and Placental (P)-cadherin, as well as Fat (ft)-cadherin.

## 2. Metabolic Rewiring During Cancer Progression

Under aerobic conditions, non-proliferating differentiated cells use oxidative phosphorylation (OXPHOS) as their main energy source. However, in a phenomenon called the Warburg effect, cancer cells perform high aerobic glycolysis despite OXPHOS, which makes them metabolically distinct from normal cells [1]. The reason why cancer cells use this type of metabolism is mainly to meet high biosynthetic and bioenergetic demands, as well as to maintain their redox state. Although less efficient concerning energy production, glycolysis is a high-speed process, making it advantageous in comparison to OXPHOS metabolism. Promotion of glycolysis allows an increased diversion of the metabolic flux into different biosynthetic pathways, in order to generate building blocks to sustain active cell proliferation [1]. Additionally, it also promotes enhanced intermediate deviation to Pentose Phosphate Pathway (PPP), which is the main source of nicotinamide adenine dinucleotide phosphate (NADPH), required for reactive oxygen species (ROS) impairment, and thus for the control of cell survival and signaling [1]. As a result, this behavior contributes to cancer progression, recurrence, and therapy resistance. 

Currently, it is quite clear that the multi-step metastatic process is associated with metabolism rewiring. During the different steps of cancer progression, the adhesion between cancer cells, as well as the adhesion of cancer cells to the ECM, are continuously changing. Interestingly, it has been demonstrated that cancer-promoting cell adhesion modifications can either induce or be induced by signaling pathways intimately associated with metabolic alterations [2]. 

In early stages of cancer progression, cells increase their motility and invasion abilities, as well as degrade the ECM, in energetically costing events, involving the remodeling of cell–cell and cell–ECM interactions, rearrangements of focal adhesions, formation of invadopodia, and the activation of the Epithelial–to–Mesenchymal Transition (EMT) program [2,3]. Glycolysis plays a central role in this stage of cancer progression, since a large amount of data has established the implication of several glycolytic enzymes in the formation of invadopodia structures, cellular protrusions, with a major interference in cancer cell invasion and ECM degradation [4]. Moreover, glycolytic by-products have also been largely involved in the promotion of motility and invasive capacity of cancer cells. One example is methylglyoxal, which activates yes-associated protein (YAP) signaling and induces EMT in breast cancer cells [5]. Similarly, the final metabolite of glycolysis, lactate, promotes breast cancer progression by supporting chemoattraction and, thus, cell migration [6]. Lactate induces alterations on extracellular pH that facilitates tumor invasion, both through the destruction of adjacent normal cell populations, as well as through acid-induced degradation of the ECM and promotion of angiogenesis [7,8]. Interestingly, mitochondrial-produced ATP can also fuel the cytoskeleton remodeling and contraction required in this step of cancer progression. Mitochondrial metabolism, through the oxidation of glucose, glutamine, and fatty acids, is described to promote cancer cell invasion and metastasis [9,10,11], either through mitochondrial overload and dysfunction or through alterations in their biogenesis [12,13,14]. This effect is described to be mediated by superoxide anion, that will activate tyrosine kinases, such as Src and Pyk2, promoting cancer cell motility, as well as cell–cell and cell–matrix alterations [13,15]. Still, in breast cancer, PGC-1α (PPARγ co-activator 1α)-induced OXPHOS and mitochondrial biogenesis promote cell invasion and metastasis [14].

Cancer cells’ adhesion to the ECM through integrins leads to an increase of ROS to non-cytotoxic levels, which induce molecular signaling that ultimately modulates invasion and spreading [15,16]. However, when the attachment to ECM is lost, either in normal or in cancer cells, oxidative stress is achieved due to the higher increase of ROS to cytotoxic levels, which cause cell death [17]. Thus, after achieving the circulatory system, cancer cells need to be resistant to *anoikis* (a “loss of home” type of cell death), in order to survive in anchorage-independent conditions. So, in the absence of cell anchorage to ECM for long periods, like in systemic metastatic dissemination, cancer cells renovate their metabolism into a program that increases anti-oxidant defenses, in order to compensate the oxidative stress. This effect dictates the survival of cancer cells in circulation and promotes the establishment of metastasis [18]. This type of metabolism is achieved by the shift to glycolysis, mainly due to the diversion of intermediate metabolites to the PPP, leading to the production of NADPH, essential for the generation of a major ROS scavenger, the reduced GSH [2]. 

Finally, the establishment of micrometastasis and the formation of secondary tumors in distant organs, also requires the establishment of cell–matrix interactions, ECM remodeling, cell–cell adhesion and outgrowth and, in this way, the activation of different metabolic programs that will lead to substantial ATP production [2]. In this case, the environment of the distant organ of metastasis will guide the metabolic behavior of cancer cells [19,20,21,22,23,24,25,26].

## 3. EMT, Cancer Stemness, and Metabolic Plasticity

Currently, there is an increased recognition that EMT and cancer stemness are driven by metabolic alterations. Breast cancer stem cells (BCSCs) change their phenotype and molecular signature to survive in all different environments along the metastatic process. Thus, these cells need high levels of plasticity, driven by EMT/Mesenchymal Epithelial Transition (MET) dynamics, where EMT promotes invasion and dissemination, and MET stimulates proliferation and metastatic colonization [27,28,29,30]. In this way, BCSCs transit between two main states: a quiescent and invasive CD44^+^/CD24^−/low^ population, with an EMT signature, named EMT-BCSC; and a proliferative and epithelial-like ALDH^+^ population, the MET-BCSC [27]. Importantly, metabolism and oxidative stress were recently implicated in the transition between both BCSC phenotypes, mainly through the activation of the AMPK/HIF1α axis (AMP-activated protein kinase/Hipoxia Inducible Factor-1α). Luo *et al.* showed that EMT- and MET-BCSC populations rely on distinct metabolic pathways, having different sensitivities to glycolytic and redox inhibitors [31]. They demonstrated that glycolysis enhancement, oxidative stress and hypoxia promote the transition from a ROS-low EMT-BCSC to a ROS-high MET-BCSC state, which can be reversed by antioxidants, such as NAC (N-acetyl Cysteine). Moreover, MET-BCSCs have an increased oxidative metabolism, as well as an increased NRF2-mediated antioxidant response. Finally, it has been also demonstrated that co-targeting these two cell populations against both metabolic properties would be of powerful therapeutic value to suppress tumor growth, tumor-initiating potential, and metastasis in breast cancer [31]. 

Thus, metabolic activity dictates the EMT/MET plasticity that BCSC need for successful cancer progression and metastasis. Moreover, exploiting these metabolic vulnerabilities of distinct BCSC states provides a novel therapeutic approach to target these critical cancer cell populations.

## 4. Biomechanics, Tissue Stiffness, and Energetic Needs Regulate Cancer Cell Metabolism

During cancer progression, cancer cells are under distinct physical forces and acquire different shapes while invade the surrounding tissues, cross the endothelial barrier to enter into circulation, as well as while exit and establish metastases in distant organs. Among these forces, there are compression, shear stress, stretching, and internal tension, which lead to intense modifications of tissue architecture. Cells respond to these forces with the reinforcement of cell–cell and/or cell–matrix interactions through surface adhesion receptors. Biomechanical response involves the activation of molecular signaling that increases internal contractile forces, reorganization of the actin cytoskeleton, and cell stiffening, determining the success of cancer cell invasion. Actually, it was recently demonstrated that epithelial cells undergo a stiffening state prior to acquiring malignant features, which are usually associated with cell-softening characteristics [32].

Currently, there is an understanding concerning the connection between cell mechanics and tissue stiffness with cell metabolism, where glycolysis has a privileged role in this link. This synergy opens the possibility of combination therapies targeting both functions simultaneously and, thus, halting disease progression in a more effective way. Common oncogenic signaling pathways integrating energy-producing metabolism and energy-consuming cell’s physical and phenotypical properties, synchronize glycolysis with the cytoskeleton remodeling dynamics. For instance, the PI(3)K pathway influences cell movement [33], as well as matrix stiffness, via integrin-mediated activation of FAK (Focal Adhesion Kinase) [34,35,36,37]. Additionally, this pathway potentiates glucose uptake, by the upregulation of glucose transporters GLUT1 and GLUT4, hexokinase (HK) and stimulating phosphofructokinase (PFK) activity [38,39]. Moreover, Hu *et al*. showed that full activation of glycolysis by PI3K requires AKT activation and Rac-dependent actin remodeling, leading to an increased aldolase activity and, consequently, to an increase of the glycolytic flux [40]. 

Matrix stiffness is also associated with increased cancer agressiveness via integrin activation of β-catenin and MYC [41], that are also known to regulate cell metabolism [42]. Goo *et al.* demonstrated that this association was being made through FAK/PI3K/AKT pathway, as showed by the increase of mitochondrial biogenesis through AKT/mTOR activation of 4EBP1 [43]. Genetic defects in mitochondrial respiration and increases in glycolysis also activate AKT signaling via an increase in the metabolic potential mediated by NADH, which promotes the recruitment of GLUTs to the cell surface and increases glycolysis [44]. Still, another study on mechanotransduction involving metabolic traits showed that Rac1b forms a complex with NADPH oxidase, increasing ROS levels and Snail expression, as well as EMT in mammary epithelial cells [45]. 

Biomechanical response of cells during cancer progression is energetically expensive and requires nearly 50% of the total cellular ATP [46,47]. Energy homeostasis is controlled by AMPK; thus, when cells sensor energy crisis, activation of AMPK upregulates ATP-producing catabolism, such as FAO (Fatty Acid Oxidation) and glucose uptake, and inhibits energy-consuming processes, such as fatty acid, cholesterol, glycogen, and protein synthesis [48]. The energy needed for the increase of cell stiffness is generated mainly by AMPK-activated glucose uptake and oxidation [49]. Thus, AMPK senses energy needs for actin reinforcement at cell—cell and cell–matrix junctions along cancer progression. 

Besides its role as an energetic homeostasis effector, AMPK is also implicated in cell growth, maintenance of apico-basal polarity, impairment of ECM degradation, autophagy, mitosis, and transcription [50,51,52]. AMPK is a component of the integrin adhesome [53] and a modulator of integrin-mediated events [54,55,56,57], usually located at the leading edge of migrating cells, at lamellipodia, where increased levels of mitochondria and mitochondrial-derived ATP can be found, when compared to the cell body [54]. The increased ATP levels are followed by a significantly lower ATP:ADP ratio, leading to a local increase of mitochondrial flux, ATP levels, and cytoskeletal rearrangement [58]. Concerning molecular regulation, AMPK is activated upstream through large kinase B1 (LKB1) [59], a tumor suppressor involved in metabolism and cell proliferation, growth, and polarity [60], another indicator of the link between cellular structure and metabolism. Moreover, Schaffer *et al*. identified several AMPK substrates as adhesion and invasion related proteins [52]. Importantly, AMPK is described to positively modulate actin dynamics and protrusive events that occur in actively adhering and migrating cells, through the regulation of epithelial tight junction assembly and disassembly [51]. Still, AMPK phosphorylation is accompanied by activation of the RhoA-myosin II pathway activation [49]. However, other studies report an inhibitory role of AMPK in integrins and cell migration [61,62,63]. For example, Georgiadou *et al.* showed that AMPK is an inhibitor of β1-integrin activity through the regulation of tensin levels in fibroblasts, thus being a mediator of the inhibition of cell spreading, traction stress, and ECM assembly [61]. 

## 5. Alterations in Cell-Matrix Adhesion Modulates Metabolic Pathways

Integrins play a role in metabolic alterations in cancer. Winograd-Katz’s in silico work revealed that integrin adhesome genes are associated with several diseases, such as cancer, cardiovascular and immunological diseases, and neurological conditions, as well as with metabolic diseases [53]. 

Detachment of normal and cancer cells from ECM strongly influences cell metabolism, namely by reducing glycolytic flux and PPP, as well as mitochondrial metabolism, and consequently decreasing ATP and NADPH production, FAO and increasing ROS levels. One of the mechanisms described to be responsible for this effect is mediated by AMPK [64]. Jeon *et al*. showed that cell–ECM detachment induces glucose uptake decrease, activates LKB1, which increases AMPK activity, inhibiting acetyl-CoA carboxylases 1 and 2 (ACC1 and ACC2, respectively) that, by one side, lowers NADPH consumption in FAS (fatty acid synthesis), but by the other side, increases NADPH generation through FAO fueling [64]. Thus, in an energetic crisis induced by ECM detachment, AMPK is responsible for NADPH maintenance and for the survival of cancer cells.

Additionally, metabolic modulation induced by PDK4 (Pyruvate Dehydrogenase kinase 4) was also demonstrated to induce anoikis resistance upon ECM detachment of human mammary cells [65]. Kamarajugadda *et al.* showed an increase of PDK4 expression after ECM detachment, leading to a reduction of mitochondrial respiration, ATP production, and a stimulation of the glycolytic flux [65]. Still, Schaffer *et al.* also demonstrated this effect in epithelial cells showing a metabolic response involving antioxidant repair of the oxidative stress as being responsible for cell survival after the disconnection to cancer ECM [18]. In this work, the authors showed that ECM detachment induces loss of glucose transport and ATP deficiency, which can be rescued through overexpression of HER2, repairing glucose uptake through stabilization of EGFR and PI3K activation, via antioxidant-generating PPP. 

## 6. Modifications in Cell–Cell Adhesion Affects Cancer Metabolism

Cadherins are the major contributors to cell–cell adhesion in epithelial tissues. During embryo development, these molecules are important morphogenic and differentiation players while, in adult tissues, they are responsible for the maintenance of cell polarity, tissue integrity and homeostasis [66]. In cancer, cadherins dysfunction is usually involved in initiation, progression, as well as in metastatic processes in a wide variety of carcinomas. Alterations in their expression, resulting from genetic and epigenetic events, lead to tissue disorder, cell de-differentiation, increased cell invasion capacity, and, ultimately, to metastasis. 

**Figure 1:**
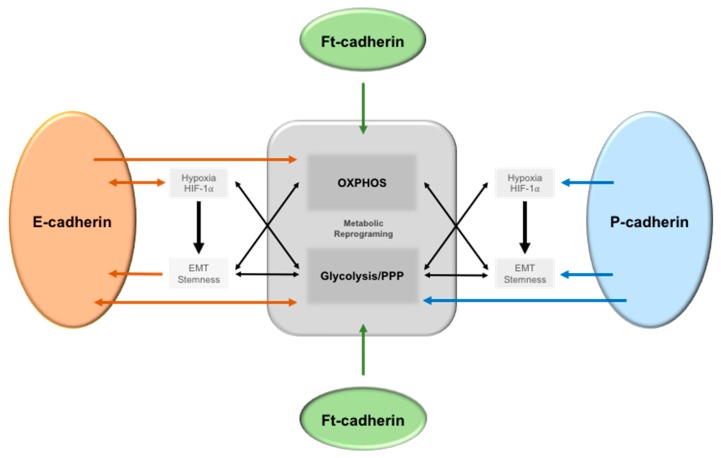
The crosstalk between E-, P-, and Ft-cadherin signaling and metabolic reprogramming in cancer cells. Tumor microenvironmental alterations, such as hypoxia, induce Epithelial–Mesenchymal Transition (EMT) and stem-like features in cancer cells via HIF-1α activation, leading to an increase in glycolysis. Additionally, increased glycolysis also induces a stem and EMT phenotype, as well as a decreased E-cadherin expression. On the other hand, E-cadherin expression can also promote oxidative phosphorylation (OXPHOS) in different cancer cell models. In relation to P-cadherin expression, it is a putative EMT marker, induces stem-like properties in cancer cells, and is associated with a hypoxic, glycolytic, and acid resistance phenotype in breast cancer. Moreover, HIF-1α induces its membrane expression, which is enriched in glycolytic BCSC. Finally, ft-cadherin is known to both enhance and inhibit OXPHOS in different models.

Interestingly, genome-wide analysis for proteins and pathways targeting Ca^2+^-dependent cell–cell adhesion identified a set of genes allocated to regulatory protein hubs according to their functions, which included metabolic processes [67]. However, knowledge about cell–cell adhesion signaling and cell metabolism is still limited and restricted mainly to few metabolic pathways. Figure 1 summarizes the associations described in the literature of the link between E-, P-, and Fat cadherins with metabolic alterations.

### 6.1. Epithelial-Cadherin (E-Cadherin)

Epithelial-cadherin, a type I classical cadherin, is a key component in the formation of adherens junctions in epithelial tissues [68]. Over the years, a huge amount of data documented E-cadherin’s role as a tumor invasion suppressor in cancer. E-cadherin expression induces an epithelioid and well-differentiated phenotype, low invasive capacity, and functional cell–cell junctions [66]. However, decreased or loss of function of this protein leads to a mesenchymal and less differentiated phenotype, increase of cell migration and invasion of the neighboring tissues, and, ultimately, to metastasis [66]. In breast cancer, loss of E-cadherin is the hallmark of lobular carcinomas [69], whereas *CDH1*/E-cadherin mutations are the initiation event of hereditary diffuse gastric cancer [66,70].

E-cadherin is the most well-studied adhesion molecule, including its association with cell metabolism summarized in Figure 1. Specific metabolic programs and microenvironment conditions induce alterations in E-cadherin expression in several types of cancer, leading to the induction of EMT [71]. Loss of E-cadherin is one of the main molecular features of EMT and is accompanied by the acquisition of cell migratory ability [72]. A large amount of data has implicated cell metabolism, through carbohydrate, amino acid, and lipid metabolism, in the control of EMT [3]. Although there is still no clear evidence about the glycolytic or OXPHOS dependency of EMT-committed cancer cells, glycolysis is the most well described pathway that impacts EMT. Hypoxia, via HIF-1α induction, as well as increased expression of glucose and monocarboxylate transporters (MCTs), activation of glycolytic enzymes, lactate secretion, and extracellular acidity, are major drivers of EMT [3]. Interestingly, Dong *et al*. demonstrated that a glycolytic phenotype induced by the loss of FBP1 is a critical oncogenic event in basal-like breast cancer, inducing BCSC and EMT phenotypes [10]. The loss of FBP1 induces glycolysis, macromolecule biosynthesis, and maintenance of ATP production under hypoxia, as well as inhibition of oxygen consumption and ROS production. This metabolic reprogramming results in an increase of stem-like properties and tumorigenicity [73]. On the other hand, EMT signaling also mediates metabolic reprogramming of cancer cells towards an increase of glucose metabolism, through increased glycolysis and PPP [74,75]. However, the role of E-cadherin in the metabolic behavior of cancer cells is far from being clearly understood and is strongly dependent on the cancer model and on the tumor microenvironment. In contrast with the above described, concerning hypoxia/HIF-1α induction of a glycolytic switch that leads to E-cadherin repression through EMT [76], there have been some recent reports demonstrating an opposite relation. Chu and co-workers demonstrated, for the first time, that E-cadherin expression is implicated in the molecular response to hypoxia and with the increase of the glycolytic behavior of cancer cells using *in vitro* and *in vivo* inflammatory breast cancer models [77]. Specifically, they showed that loss of E-cadherin and/or overexpression of its repressors, such as ZEB1, downregulates the expression of HIF-1α and CAIX, leading to a reduction of the extracellular acidification of inflammatory breast cancer cells, tumor growth, and metastasis formation [77]. 

Cell–cell adhesion though E-cadherin can also be crucial to anoikis resistance of cells when detached from the ECM. Bergin *et al.* demonstrated that E-cadherin is responsible for resistance to anoikis of mouse proximal tubular aggregates detached from ECM, in a PI3K dependent manner [78]. Moreover, in oral squamous cell carcinomas, E-cadherin regulates anchorage-independent growth and survival [79]. Recently, E-cadherin has been also described as a regulator of energy metabolism, making the link between energetic balance and mechanotransduction in different cancer models. Park and co-workers demonstrated that E-cadherin is able to maintain cell proliferation by regulating energy metabolism through NF-κB [80]. Induced E-cadherin expression led to an increase of ATP levels through the increase of OXPHOS and glycolysis, as well as to an increase in ROS production. Moreover, the induction of this cadherin led to NF-κB mediated increase of glucose transport through GLUT1 expression, shifting from glutamine to glucose dependency [80]. Moreover, E-cadherin is also required to stimulate AMPK activation. Bays and co-workers demonstrated that application of shear stress to epithelial monolayers, or application of force directly to E-cadherin, leads to the stimulation of AMPK, through LKB1 recruitment to the E-cadherin mechanotransduction complex, leading to RhoA-mediated contractibility and reinforcement of the actin cytoskeleton [49]. Still, Sebbagh *et al.* demonstrated that LKB1/STRAD complex co-localizes with E-cadherin at adherens junctions and that this cadherin controls AMPK phosphorylation by controlling LKB1 localization in polarized epithelial cells [81].

There are still other works establishing a link between AMPK and E-cadherin, through the inhibition of EMT in several cellular models [82,83,84]. In an energetic point of view, Bays *et al.* identified glycolysis as the main ATP producing path to the formation and maintenance of an epithelial barrier [49]. However, detailed information on glycolysis-escaping pathways, such as PPP or serine/ glycine synthesis, are needed in order to confirm the exact ATP-producing step. 

Finally, E-cadherin has also been associated with other metabolic players of cancer cells, such as PKM2, a key enzyme of the glycolytic pathway, where PKM2 silencing induced the upregulation of caspase 7, Bad and E-cadherin expression, as well as to a decrease of MMP (Matrix Metalloproteinase) 2 and 9, HIF1α, and VEGF, impairing cell migration and invasion, inhibiting proliferation, and inducing apoptosis and cell cycle arrest at the G0/G1 phase [85]. 

### 6.2. Placental Cadherin (P-Cadherin)

P-cadherin (or placental cadherin) is a classical cell–cell adhesion molecule, encoded by the *CDH3* gene, whose expression is highly associated with undifferentiated cells in normal adult epithelial tissues, as well as with poorly differentiated carcinomas. In breast cancer, P-cadherin is *de novo* expressed in approximately 30–40% of invasive carcinomas and is a poor prognostic factor in this disease. Its expression is significantly associated with tumors of high histological grade and short-term overall and disease-free survival, as well as with a distant and loco-regional relapse-free interval [86,87,88,89]. Interestingly, P-cadherin is a marker of heterogeneous and worse prognosis triple-negative basal-like breast carcinomas [86,90], described to present a more glycolytic behavior by their increased expression of glycolytic markers [91].

To the best of our knowledge, the link between cancer cell metabolism and P-cadherin-mediated adhesion was made with our work in 2014, where we suggested a role for this epithelial basal marker in cancer cell metabolism [91] (Figure 1). In this study, we established an association between aberrant P-cadherin expression and hypoxic, glycolytic and acid-resistant breast cancer cells. 

Over the years, our group has been studying the mechanisms behind P-cadherin induced-aggressive behavior in cancer cells in the different steps of breast cancer progression [92,93,94]. We showed that P-cadherin promotes cancer cell invasion, enhancing the initial stages of breast cancer progression [95,96]. Mechanistically, P-cadherin overexpression induces the secretion of MMPs, which degrade the ECM [95,96] and also cleave its extracellular domain to produce a soluble P-cadherin fragment (sP-cad) with pro-invasive capacity [95]. Importantly, we demonstrated that P-cadherin aberrant expression was significantly associated with the expression of HIF-1α, GLUT1, CAIX, MCT1, and CD147 in human primary invasive breast tumors, which is in line with the glycolytic phenotype of P-cadherin-enriched basal-like breast carcinomas [91]. Moreover, we also showed that *CDH3* silencing led to a decrease of the mRNA levels of GLUT1 and CAIX in breast cancer cell lines in vitro [91]. Thus, considering that acidic pH conditions induces the proteolytic activity of cancer cells [97,98], the increased glycolytic behavior and the consequent ECM acidification might also be responsible for the increased proteolytic activity of P-cadherin enriched breast cancer cells. 

The aggressive cancer cell behavior induced by this molecule is also due to its biomechanical properties [99]. In a wild type E-cadherin context, P-cadherin have a pronounced influence on the actin cytoskeleton, since it induces the destabilization of the membrane E-cadherin/p120-catenin complex, leading to the delocalization of β-catenin and p120-catenin to the cytoplasm, modifying the actin cytoskeleton polymerization, promoting invasion, migration and motility, as well as tumorigenic potential [100,101,102,103]. P-cadherin also induces phosphorylation of Src-family kinases (SFK) and FAK, as well as the activation of Rac1 small GTPase [101]. Recently, we also demonstrated that P-cadherin/SFK signaling induces topological, morphological, and biomechanical alterations [99], by the modulating cellular height and area and intercellular organization, as well as by decreasing cell–cell adhesion and stiffness [99]. Moreover, Trepat’s group identified different mechanical roles for both E- and P-cadherins, showing that P-cadherin predicts levels of intercellular force, while E-cadherin predicts the rate at which intercellular force builds up [104]. Still, P-cadherin enhances adhesion of breast cancer cell to ECM through the activation of the heterodimer α6β4 integrin, which promotes the binding of cancer cells specifically to laminin in the ECM [100]. 

Thus, considering that P-cadherin aberrant expression induces a phenotype to breast cancer cells that promotes the initial stages of cancer progression, mainly through energy-costing processes, this would be probably assisted by the enhancement of energy producing metabolic pathways, such as oxidative phosphorylation and/or enhanced glycolysis. 

Finally, P-cadherin is also associated with successful breast cancer metastasis. Its expression shows a strong correlation with invasion of vascular and soft tissues [105]. Moreover, our group have identified P-cadherin as an independent indicator of prognosis in the metastatic setting of breast cancer patients, as well as a putative useful biomarker for axillary-based breast cancer decisions in clinical practice [106]. This metastatic promoter role is probably due to its role in maintaining cancer stem-like characteristics, promoting cell survival in the circulatory system. P-cadherin-enriched breast cancer cell populations comprised increased *in vitro* mammosphere-forming efficiency, and its expression promotes resistance to anoikis [107]. Still, P-cadherin provides resistance to x-ray-induced cell death, implicating this molecule in another cancer stem cell property [107]. Importantly, BCSC fractions harboring high levels of P-cadherin showed also concomitant expression of HIF-1α, CAIX, and GLUT1 [91], reinforcing the glycolytic behavior of P-cadherin enriched BCSC populations.

In breast cancer, hypoxia induces stem-like properties, as well as expansion of BCSC and EMT [108]. Interestingly, we demonstrated that nuclear HIF-1α is able to induce P-cadherin expression at the cell membrane [91]. Recently, an overview of P-cadherin in EMT process associates this molecule with an “intermediate/metastable” phenotype, suggesting P-cadherin as a putative EMT marker and supporting the use of anti-P-cadherin therapeutics to metastatic breast cancer [92]. Importantly, P-cadherin identifies an intermediate state between the epithelial and the mesenchymal phenotypes. 

### 6.3. Fat (ft)-like Cadherin

Fat (ft) cadherin is a *Drosophila* protocadherin, with an extracellular domain of 34 cadherin repeats and a large intracellular domain, including EGF-like and laminin-G-like domains [109]. This protein can be expressed at lamellipodia, filopodia, and at cell–cell contacts, and, similarly to classical cadherins, plays a role in cell adhesion and signaling. Ft is known to impair cellular growth, acting upstream the Hippo signaling pathway and with a conserved function during planar cell polarity establishment [110,111].

There are four human orthologues of *Drosophila* FAT, FAT1-4, and the closest related one is FAT4 [112]. Alterations, such as mutations and deletions in FAT family, have been described in several types of human cancer [113]. However, these genes exhibit a dual role as either tumor suppressor genes (TSG) [113,114] or oncogenes [115,116,117,118], depending on the cellular context. Molecularly, ft are described to signal through some canonical pathways in cancer cells, such as Wnt/β-catenin and MAPK/ERK [119].

Interestingly, ft can also induce cell metabolic alterations (Figure 1). In 2014, Sing and co-workers elegantly demonstrated a direct role of ft in mitochondrial morphology and function [120]. These authors showed that ft controls ROS production, stabilizes the mitochondrial ETC (Electron Transport Chain), and promotes oxidative phosphorylation during *Drosophila* development. This effect is mediated by the activity of a 68 KDa soluble fragment, ft^mito^, generated by the proteolytic cleavage of ft, which is transported to the mitochondria to bind Ndufv2 (NADH dehydrogenase ubiquinone flavo-protein 2), promoting complex I and V assembly and stability, and enhancing mitochondrial ETC [120]. 

Ft is usually located at the cell membrane and is, only in this location, able to impair proliferation through the activation of Hippo kinase pathway. Thus, ft cleavage and delocalization from membrane to the mitochondria no longer impairs proliferation and promotes OXPHOS metabolic behavior. These authors propose a model for ft mutants, such as cancer cells, where both PCP and Hippo are dysregulated, OXPHOS is impaired, and there is an increase of ROS and a metabolic shift towards glycolysis, with increased ATP and lactate production [120]. Moreover, Cao *et al*. showed that atypical Fat1 cadherin acts as a molecular ‘brake’ on mitochondrial respiration, regulating vascular smooth muscle cell (SMC) proliferation after arterial injury, using *in vitro* and *in vivo* models [121]. These authors showed that SMCs lacking Fat1 consume more oxygen for ATP production and contain more aspartate, while the introduction of a modified Fat1 intracellular domain normalizes the oxygen consumption as well the SMC growth ability [121]. Specifically, Fat1 deletion increases the activity of mitochondrial complexes I and II, mediating growth control intrinsically through the mitochondria [121]. 

However, the role of ft in cancer cell growth through its activity in metabolism is far from being understood. To the best of our knowledge, there are only a couple of works showing this direct link. Madan *et al.* reported the existence of a FAT1-HIF-1α axis in glioblastoma, responsible for the agressiveness of this disease and representing a new potential therapeutic target for these tumors [122]. These authors showed that FAT1 is associated with HIF-1α expression, as well as with its targets PGK1 (Phosphoglycerate kinase 1) and VEGFA in human glioblastoma samples. *In vitro*, they demonstrated that severe hypoxia induces the expression of FAT1 and that the depletion of endogenous FAT1 under hypoxia induces a decrease in HIF-1α, CAIX, GLUT1, VEGFA, MCT4, HK2, BNIP3, and REDD1, as well as a decrease in glioblastoma cell invasion [122].

Thus, based on what is reported in the literature, it is reasonable to speculate that the loss of ft cadherins will promote a mitochondrial dysfunction and a metabolic shift in cancer cells. However, further studies are still needed to understand the metabolic impact of ft cadherins alterations in cancer cells.

## 7. Concluding Remarks

**Figure 2:**
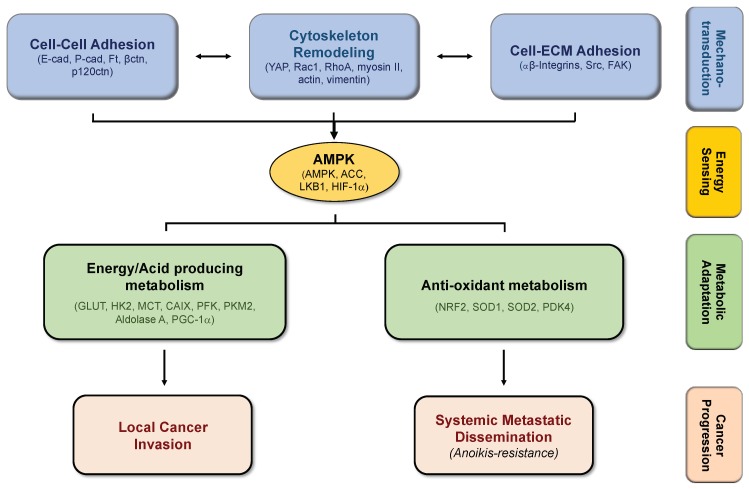
The molecular link between mechanotransduction and metabolic adaptations promotes cancer cell progression. Alterations in cell–cell and cell–ECM adhesion, leading to cytoskeleton remodeling, requires AMPK-induced ATP production through specific metabolic programs, supporting cancer progression.

During cancer progression, cells need to adapt in order to successfully accomplish each barrier, from the primary tumor until the formation of metastasis. Alterations in cell–cell adhesion, as well as on cell-ECM interactions, that further stimulate cytoskeleton remodeling, are stimuli that induce the mechanotransduction of cancer cells to active signaling pathways, allowing them to transduce their morphological and survival needs. Alterations on cancer cells’ mechanotransduction require energy, which is sensored by AMPK. This important molecular player activates different metabolic processes, in order to meet the specific energetic demands that cancer cells need in the different steps of cancer progression, sustaining proliferation and survival of cancer cells, and thus promoting cancer progression (Figure 2). 

## Abbreviations

ACC1/ACC2	acetyl-CoA carboxylase 1/2
ADP	Adenosine diphosphate
AKT	Protein kinase B (PKB)
ALDH	Aldehyde Dehydrogenase
AMPK	AMP-activated protein kinase
ATP	Adenosine triphosphate
Bad	BCL2 Associated Agonist of Cell Death
BCSC	Breast Cancer Stem Cells
BNIP3	BCL2 Interacting Protein 3
CAIX	Carbonic Anhydrase IX
CD147	Cluster of Differentiation 147
E-cadherin	Epithelial cadherin
ECM	Extracellular Matrix
EGF	Epidermal Growth Factor
EGFR	Epidermal Growth Factor Receptor
EMT	Epithelial Mesenchymal Transition
ERK	Extracellular-signal-Regulated Kinase
ETC	Electron Transport Chain
FAK	Focal Adhesion Kinase
FAO	Fatty Acid Oxidation
FAS	Fatty Acid Synthesis
FBP1	Fructose-1,6-bisphosphatase 1
Ft	Fat
Ftmito	Mitochondrial Fat Soluble fragment
GLUT1/4	Glucose Transporter ¼
GTP	Guanosine-5′-triphosphate
HER2	Human Epidermal Growth Factor Receptor 2
HIF-1α	Hipoxia Inducible Factor-1α
HK2	Hexokinase 2
LKB1	Large Kinase B1
MAPK	Mitogen-Activated Protein Kinase
MCT	Monocarboxylate Transporters
MET	Mesenchymal Epithelial Transition
MMP	Matrix Metalloproteinase
mTOR	mammalian target of rapamycin
MYC	*Myelocytomatosis* oncogene
NAC	N-acetyl Cysteine
NADPH	Nicotinamide Adenine Dinucleotide Phosphate
Ndufv2	NADH dehydrogenase ubiquinone flavo-protein 2
NF-κB	Nuclear Factor Kappa B
OXPHOS	Oxidative Phosphorylation
P-cadherin	Placental-cadherin
PDK4	Pyruvate Dehydrogenase kinase 4
PFK	Phosphofructokinase
PGC-1α	PPARg co-activator 1α
PGK1	Phosphoglycerate kinase 1
PI(3)K	Phosphoinositide 3-kinase
PKM2	Pyruvate Kinase isozyme Muscle 1
PPARγ	Peroxisome proliferator-activated receptor gamma
PPP	Pentose Phosphate Pathway
Rac	Ras-related C3 botulinum toxin substrate 1
REDD1	Regulated in Development and DNA Damage Response 1
ROS	Reactive Oxygen Species
SFK	Src family Kinase
SMC	smooth muscle cell
sP-cad	soluble P-cadherin fragment
STRAD	STe20 Related ADapter
TSG	Tumor Suppressor Gene
VEGF	Vascular Endothelial Growth Factor
YAP	Yes-associated protein
